# Tracking peripheral vascular function for six months in young adults following SARS‐CoV‐2 infection

**DOI:** 10.14814/phy2.15552

**Published:** 2022-12-21

**Authors:** Valesha M. Province, Rachel E. Szeghy, Nina L. Stute, Marc A. Augenreich, Christian E. Behrens, Jonathon L. Stickford, Abigail S. L. Stickford, Stephen M. Ratchford

**Affiliations:** ^1^ Department of Health & Exercise Science Appalachian State University Boone North Carolina USA

**Keywords:** COVID‐19, flow‐mediated dilation, inflammation, oxidative stress, nitric oxide, passive limb movement

## Abstract

SARS‐CoV‐2 infection is known to instigate a range of physiologic perturbations, including vascular dysfunction. However, little work has concluded how long these effects may last, especially among young adults with mild symptoms. To determine potential recovery from acute vascular dysfunction in young adults (8 M/8F, 21 ± 1 yr, 23.5 ± 3.1 kg⋅m^−2^), we longitudinally tracked brachial artery flow‐mediated dilation (FMD) and reactive hyperemia (RH) in the arm and hyperemic response to passive limb movement (PLM) in the leg, with Doppler ultrasound, as well as circulating biomarkers of inflammation (interleukin‐6, C‐reactive protein), oxidative stress (thiobarbituric acid reactive substances, protein carbonyl), antioxidant capacity (superoxide dismutase), and nitric oxide bioavailability (nitrite) monthly for a 6‐month period post‐SARS‐CoV‐2 infection. FMD, as a marker of macrovascular function, improved from month 1 (3.06 ± 1.39%) to month 6 (6.60 ± 2.07%; *p* < 0.001). FMD/Shear improved from month one (0.10 ± 0.06 AU) to month six (0.18 ± 0.70 AU; *p* = 0.002). RH in the arm and PLM in the leg, as markers of microvascular function, did not change during the 6 months (*p* > 0.05). Circulating markers of inflammation, oxidative stress, antioxidant capacity, and nitric oxide bioavailability did not change during the 6 months (*p* > 0.05). Together, these results suggest some improvements in macrovascular, but not microvascular function, over 6 months following SARS‐CoV‐2 infection. The data also suggest persistent ramifications for cardiovascular health among those recovering from mild illness and among young, otherwise healthy adults with SARS‐CoV‐2.

## Graphical abstract

Brachial artery flow‐mediated dilation, a marker of macrovascular function, increased from months 1–6 following SARS‐CoV‐2 infection without alteration to reactive hyperemia in the arm or leg, markers of microvascular function, and circulating biomarkers. These findings suggest a prolonged time course for macrovascular recovery following SARS‐CoV‐2 infection in young adults which is important for understanding the persistent ramifications of SARS‐CoV‐2 on cardiovascular health.

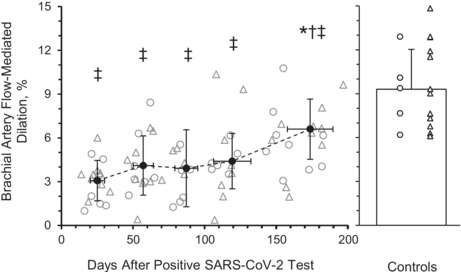

## 1 INTRODUCTION

The novel coronavirus disease of 2019 (COVID‐19) has been a major burden on worldwide healthcare and daily life for over two years. The severe acute respiratory syndrome coronavirus‐2 (SARS‐CoV‐2), responsible for the COVID‐19 pandemic, has infected over 320 million and caused over 5.5 million deaths worldwide by January 2022 (Dong et al., 2020). SARS‐CoV‐2 can inhibit angiotensin‐converting enzyme‐2 (ACE2) receptor activity (Zhang et al., 2020), as well as elicit systemic inflammation via a cytokine storm (Barnes et al., 2020; Paybast et al., 2020). Early investigations revealed SARS‐CoV‐2 is able to infect and cause inflammation of vascular endothelial cells and directly impact vascular function and blood flow regulation (Bhagat & Vallance, 1997; Hingorani et al., 2000; Varga et al., 2020), which can have significant systemic ramifications (Nakano et al., 2021; Paybast et al., 2020). This cascade of events may cause functional decrements seen throughout the vasculature, including vascular dysfunction among young (Nandadeva et al., 2021; Ratchford et al., 2020) and old adults (Ambrosino et al., 2021; Ergul et al., 2021; Lambadiari et al., 2021; Paneroni et al., 2021; Riou et al., 2021). Yet, to date, little is known regarding the long‐term impact of the SARS‐CoV‐2 infection on the vasculature, especially among those with mild symptoms who may be overlooked or expected to not have long‐term consequences.

While the severity of vascular consequences caused by SARS‐CoV‐2 infection is unclear, current studies focusing on vascular function have been cross‐sectional in nature and have failed to establish a timeline following SARS‐CoV‐2 infection of vascular health and recovery among adults. Our group has previously observed significant vascular deficits acutely among young adults with SARS‐CoV‐2 (Ratchford et al., 2020), evidenced by decreases in brachial artery flow‐mediated dilation (FMD), as an indication of macrovascular function that is highly correlated with coronary artery function (Thijssen et al., 2011, 2019). In addition, a blunted reactive hyperemic (RH) response to single passive limb movement (sPLM), an indication of muscle microvascular dysfunction, was observed among these young adults 3 to 4 weeks after SARS‐CoV‐2 infection (Ratchford et al., 2020). These results were corroborated by others who identified a blunted hyperemic response to sPLM, in hospitalized individuals 2 to 4 months after SARS‐CoV‐2 infection (Paneroni et al., 2021). This blunted hyperemic response has even been observed during continuous PLM (cPLM) prior to SARS‐CoV‐2 symptom onset (Trinity et al., 2021). These results may suggest hypersensitive afferent and central hemodynamic responses to cPLM during acute infection and inflammatory responses such as during SARS‐CoV‐2 (Fudim et al., 2020). Further, lower FMD and RH in symptomatic young adults 1 to 4 months after infection with SARS‐CoV‐2 compared with healthy young adults (Nandadeva et al., 2021) may represent a prolonged disruption in vascular function. Likewise, middle‐aged and older adults displayed reduced FMD compared with healthy controls at 2 (Ambrosino et al., 2021) and 3 (Riou et al., 2021) months following hospitalization, as well as at 4 months post‐infection (Lambadiari et al., 2021). Despite growing evidence of vascular dysfunction among those infected with SARS‐CoV‐2, these findings lack a time‐dependent association with symptom severity as individuals recover from SARS‐CoV‐2.

The cytokine storm caused by SARS‐CoV‐2 instigates systemic inflammation (Barnes et al., 2020; Paybast et al., 2020), which is known to affect vascular function. Markers of inflammation, such as interleukin‐6 (IL‐6) and C‐reactive protein (CRP), are strong predictors of the presence and severity of SARS‐CoV‐2 infection (Coomes & Haghbayan, 2020; Kermali et al., 2020), as well as predictors of the risk of cardiovascular events (Avan et al., 2018; Ridker et al., 2000), and mortality (Ruan et al., 2020). Similarly, SARS‐CoV‐2 infection can cause an increase in mitochondrial reactive oxygen species (ROS), evoking increases in oxidative stress and inflammation as well as decreases in bioavailability of the potent endothelial‐derived vasodilator, nitric oxide (NO) (Fedorova et al., 2014; Morris et al., 2020; Pearce et al., 2020; Saeed & Mancia, 2021). Elevated lipid peroxidation (malondialdehyde, MDA or thiobarbituric acid reactive substance, TBARS) levels have also been observed in many disease states (Mutlu‐Turkoglu et al., 2003), including SARS‐CoV‐2 for periods up to 4 months post‐infection (Lambadiari et al., 2021), suggesting persistent elevations in oxidative stress. Together, these findings suggest the potential role of several inflammatories, ROS, and endothelial‐derived biomarkers in the vascular dysfunction observed among those infected with SARS‐CoV‐2.

Thus, this investigation sought to extend our findings of vascular dysfunction in the 3 to 4 weeks following SARS‐CoV‐2 infection and examine the natural time course for potential vascular function improvements over a 6‐month period. Using a series of non‐invasive vascular assessments each month among young adults recovering from SARS‐CoV‐2, we hypothesized vascular function would improve from months one to six following SARS‐CoV‐2 infection (Carfi et al., 2020; Mahase, 2020), as evidenced by increases in macrovascular function in brachial artery FMD, microvascular function in brachial artery RH following cuff occlusion, and mobility muscle microvascular function in the femoral artery RH during PLM. As an exploratory analysis, we sought to study the afferent response via the central hemodynamic assessment to cPLM, which we hypothesized would be augmented from months one to six following SARS‐CoV‐2 infection. Additionally, we sought to explore if participants' functional outcome measures were cross‐sectionally similar to healthy control participants by the completion of the study. Finally, this study sought to better understand the long‐term effects of SARS‐CoV‐2 infection on circulating markers of inflammation, ROS, and NO bioavailability as potential mechanisms for vascular dysfunction and recovery. We hypothesized markers of inflammation (IL‐6, CRP) and ROS (TBARS, protein carbonyl) would decrease, and antioxidant capacity (superoxide dismutase, SOD) and the potent vasodilator nitrite would increase from months one to six following SARS‐CoV‐2 infection. The current study provides a comprehensive, longitudinal assessment of peripheral vascular function and potential vascular mediators that has otherwise been lacking in the literature and may provide additional guidance for cardiovascular health and recovery efforts following SARS‐CoV‐2 infection in young adults.

## 2 METHODS

### 2.1 Participants

Otherwise healthy young adults who tested positive for SARS‐CoV‐2 using a nasopharyngeal swab polymerase chain reaction (PCR) assay reported to the laboratory 3–4 weeks following their positive test and for four additional visits approximately 1 month apart. All participants were non‐smokers, free from chronic cardiovascular, metabolic, or renal disease, and female participants were premenopausal and not currently pregnant or breastfeeding. Likewise, these participants did not require hospitalization during or following infection.

As described previously (Ratchford et al., 2020), retrospective control participants were studied February 4–6, 2020 prior to the first confirmed case of COVID‐19 in North Carolina, United States on March 3, 2020 (Services NDoHaH, 2020) and prior to the World Health Organization declaring COVID‐19 a pandemic on March 11, 2020. Control subjects had not experienced flu‐like symptoms.

### 2.2 Study procedures

Testing took place in a quiet, environmentally controlled laboratory, with an ambient temperature of ~23°C. Participants returned for monthly testing to repeat the same ordered measurements. Participants arrived at the laboratory in a fasted state, having abstained from exercise, caffeine, and alcohol for at least 12 h before testing and were at least 4 h postprandial. Participants were asked to consume a low‐fat diet and minimal food intake (Padilla et al., 2006; Vogel et al., 1997) when subjects could only abstain from food for 4 h instead of the traditional 6 h set forth by recommended guidelines (Harris et al., 2010; Thijssen et al., 2011) prior to testing. Upon arrival, participants were asked to rank their current symptom severity using an in‐house survey, and anthropometric data were collected. Participants lay supine on a bed quietly for 20 min while being instrumented. Resting supine brachial artery blood pressures [systolic blood pressure (SBP); diastolic blood pressure (DBP); mean arterial pressure (MAP), as MAP = [((2/3)·DBP) + ((1/3)·SBP)]] and heart rate (HR) were obtained (Evolv, Omron Healthcare, Japan). Vascular function assessments were performed on participants. After vascular function testing, an antecubital venous blood draw was obtained. All Doppler ultrasound imaging and data processing and analysis were performed by a single, trained researcher throughout the study.

### 2.3 SARS‐CoV‐2 symptom severity survey

Participants were asked to rank their SARS‐CoV‐2 symptoms each day of testing on a symptom severity survey (SSS), as previously described (Ratchford et al., 2020). On a scale of 0–100 of increasing severity, participants ranked their symptoms of chest pain, chills, diarrhea, dizziness or vertigo, dry cough, dry eyes, dry mouth, fatigue, fever over 37.9°C, headache, lack of appetite, loss of smell or taste (anosmia), muscle or body aches, nasal congestion or runny nose, nausea or vomiting, shortness of breath, difficulty breathing, dyspnea, sore joints, or sore throat. The values for all symptoms above a score of zero were totaled and averaged for total symptom severity. Mild symptoms were categorized as a symptom severity score of 0–33, moderate from 34 to 66, and severe as a score of 67 of 100. Additionally, participants subjectively provided their recent physical activity level each month based on days each week and duration of physical activity.

### 2.4 Brachial artery flow‐mediated dilation

Participants rested in the supine position for 20 min before the start of data collection with the right arm abducted 90 °, and the elbow joint extended at heart level. Brachial artery FMD measurements were obtained from the right brachial artery using current guidelines as a functional, upper limb marker of vascular function and cardiovascular risk (Thijssen et al., 2011). Baseline measurements of the right brachial artery diameter and blood velocity were taken for 1 min using a Doppler ultrasound system (GE Logiq eR7 and L4‐12T‐RS transducer, GE Medical Systems). Sample volume was optimized in relation to vessel diameter and centered within the vessel for each participant. Measurements of brachial artery diameter and velocity were obtained with the Doppler ultrasound in duplex mode with a B‐mode imaging frequency of ~12 MHz and a Doppler frequency of approximately 4 MHz. An angle of intonation of ≤60° (Rizzo et al., 1990) was achieved for all measurements. Immediately after baseline measurements, a blood pressure cuff placed distal to the elbow, was rapidly inflated to 250 mmHg for 5 min. The blood pressure cuff was rapidly deflated, and brachial artery diameter and blood velocity were recorded for 2 min. Brachial artery diameter and blood velocity were analyzed offline for continuous second‐by‐second measurements (Cardiovascular Suite v. 4.0, Quipu). Brachial artery FMD was quantified as the maximal change in brachial artery diameter after cuff release, expressed as a percentage increase from pre‐occlusion values (%FMD). Shear rate (SR) was calculated as follows: [SR (s^−1^) = blood velocity · 8/vessel diameter]. The %FMD was made relative to baseline diameter and SR to account for daily fluctuations in baseline diameter and SR‐induced changes in vasodilation. Peak velocity and peak velocity relative to MAP were assessed to predict cardiovascular disease risk and future cardiovascular events (Huang et al., 2007).

### 2.5 Brachial artery reactive hyperemia

Brachial artery RH was simultaneously measured and determined as the 2 min area under the curve (AUC) for the BF response following cuff occlusion, providing an index of microvascular function (Harris et al., 2010), which is inversely related to cardiovascular disease risk and predicts future cardiovascular events in healthy and diseased populations (Huang et al., 2007). Cumulative values of the AUC for SR and BF were integrated via the trapezoid rule and calculated as: Σ{*yi* [x(I + 1) – *xi*] + (½)[*y*(I + 1) – *yi*][*x*(I + 1) – *xi*]}; (*x* is time, *y* is shear rate, *xi* is initial time point, *yi* is initial blood velocity; Huang et al., 2007). Blood flow (BF) was determined as: BF, ml·min^−1^ = [blood velocity · π · (arterial diameter · 2^−1^)^2^] · 60. Brachial artery BF was made relative to MAP (vascular conductance, VC) to account for daily changes in driving pressure. In pilot testing, the average coefficient of variation for 2 individuals in triplicate assessment for baseline diameter (0.7%), peak diameter (0.5%), sum of shear at peak (9.4%), %FMD (10.7%), FMD/Shear (6.8%), and RH (2.1%) was similar to previously published reproducibility assessments for FMD (Ghiadoni et al., 2012).

### 2.6 Femoral artery single passive leg movement

Femoral artery single PLM (sPLM) measurements were obtained from the right common femoral artery using current guidelines as a functional, lower‐limb assessment of microvascular function (Gifford & Richardson, 2017). While in the supine position with the participant's left leg supported on a table and right leg supported by a research team member at heart level, baseline measurements of the common femoral artery diameter and blood velocity, at least 3 cm proximal to the femoral artery bifurcation, were recorded for 1 min before performing the PLM maneuver using similar Doppler ultrasound system settings used for the brachial artery FMD procedure (B‐mode imaging frequency of ~12 MHz and Doppler frequency of ~4 MHz). Immediately following baseline measurements, the research team member supporting the right thigh and ankle manually moved the knee joint one time through a 90° range of motion, flexion extension, at 1 Hz while common femoral artery diameter and blood velocity were recorded for 1 min after the movement. Common femoral artery diameter and blood velocity were analyzed offline for second‐by‐second measurements as described above (Cardiovascular Suite v. 4.0, Quipu).

### 2.7 Femoral artery continuous passive leg movement

Following the sPLM, a cPLM assessment was performed on the same leg. The research team member manually moved the knee joint through a 90° range of motion, flexion extension, at 1 Hz continuously for 1 min, while common femoral artery diameter and blood velocity were recorded during this cPLM and 1 min AUC. Analysis was performed offline for continuous second‐by‐second measurements (Cardiovascular Suite v. 4.0, Quipu). The 1 min AUC response and VC were determined as indices of microvascular function (Gifford & Richardson, 2017). Heart rate (HR), stroke volume (SV), cardiac output (CO), and mean arterial blood pressure (MAP) were determined noninvasively using photoplethysmography (Finometer, Finapres Medical Systems BV) throughout the cPLM test to determine central hemodynamic responses to the afferent feedback elicited by the cPLM (Ives et al., 2016). Baseline, peak, change from baseline to peak (ΔPeak), and 60‐s AUC (AUC60) were determined for all variables of interest. In pilot testing, the average coefficient of variation for 3 individuals in triplicate assessments for baseline blood flow, peak blood flow, delta blood flow, and area under the curve for sPLM (2.6%, 3.8%, 18.0%, and 18.7%, respectively) and cPLM (7.3%, 12.2%, 29.6%, and 22.2%, respectively) was similar to recently published reproducibility assessments of cPLM (Groot et al., 2022; Lew et al., 2021).

### 2.8 Markers of oxidative stress, inflammation, nitric oxide bioavailability, and antioxidant levels

Venous blood draws were performed by a trained phlebotomist following vascular measurements. Plasma and serum were separated by centrifugation and stored at −80°C until analysis. Biochemical assays were performed according to the manufacturer's instructions for the oxidative stress biomarkers: TBARS (Cayman Chemical, Ann Arbor, MI, 10009055), as an indicator of MDA and lipid peroxidation, and serum protein carbonyl using a colorimetric assay (Cayman Chemical, Ann Arbor, MI, 10005020), as an indicator of protein oxidation; inflammatory biomarkers, plasma CRP (Cayman Chemical, Ann Arbor, MI, 10011236) and plasma IL‐6 using enzyme‐linked immunosorbent assays (Cayman Chemical, Ann Arbor, MI, 501030); the serum antioxidant biomarker SOD using an activity assay (Cayman Chemical, Ann Arbor, MI, 706002) and NO bioavailability, through plasma measurement of the putative endpoint, nitrite, utilizing a NO assay kit (ThermoFisher Scientific, Vienna, Austria, EMSNO).

### 2.9 Statistical analyses

Statistical analyses were performed using commercially available software (IBM SPSS Statistics Version 26, Armonk, NY; SAS Version 9.4, Cary, NC). Each continuous variable was checked for normality using Kolmogorov–Smirnov tests. Normality was confirmed visually with QQ‐plot observations. Linear mixed models were used to determine the main effects of time (monthly visits) for all outcome variables. Studentized residuals falling outside of three standard deviations would have been considered outliers and removed from analysis; however, no outliers were detected. Significant findings were determined by an alpha level of 0.05 for pairwise comparisons. Eta‐squared was measured as an indicator of the main effect size for FMD, RH, sPLM, and cPLM functional outcome measures. Tukey–Kramer post hoc correction was used where significant effects were observed, and adjusted p‐values are reported. Hedge's *g* was measured using pooled standard deviation for all the groups along with a correction factor, as an indicator of effect size for FMD, RH, sPLM, and cPLM functional outcome measures. As an exploratory analysis and to provide the context of expected healthy control data, one‐way repeated measures ANOVA with planned comparison analyses were performed between the SARS‐CoV‐2 participant visits and healthy control participants to determine group differences for FMD, RH, sPLM, and cPLM functional outcome measures. Pearson correlations were performed to determine the potential associations between the changes to functional indices of vascular function (i.e., FMD, FMD/Shear, sPLM BF AUC, or cPLM BF AUC) and the changes to circulating biomarkers (i.e., CRP, IL‐6, SOD, TBARS, protein carbonyl, and nitrite) from months one to six; these correlations may exist on an individual subject level even when group difference do not exist from month to month. For these correlations, each participant with a M1 and M6 visit was plotted as an individual data point. Values are expressed as means ± SD.

## 3 RESULTS

### 3.1 Participants

Participant characteristics (8 M/8F) can be found in Table 1. Some participant data were previously published (Ratchford et al., 2020) although additional participants and study visits were included in the current investigation. There was a main group effect between participants with SARS‐CoV‐2 and retrospective healthy control participants for age (*p* = 0.001, *η* ^2^ = 0.302) and weight (*p* = 0.015, *η* ^2^ = 0.153), but not for height (*p* = 0.055, *η* ^2^ = 0.120), BMI (*p* = 0.761, *η* ^2^ = 0.030), physical activity frequency (*p* = 0.848, *η* ^2^ = 0.023), or physical activity duration (*p* = 0.889, *η* ^2^ = 0.020; Table 1). Study attrition (due to time, availability, interest, etc.) over the six‐month period led to combining the participants' final visit (month 5 and month 6), with 12 participants completing five visits (month 1: M1, month 2: M2, month 3: M3, month 4: M4, and month 6: M6). Participant‐positive PCR tests were obtained between September 2, 2020 and January 11, 2021. Visits M1‐M4 began approximately 3–4 weeks post‐positive PCR test, and every 3–4 weeks following. M1 took place 14‐34d post‐positive PCR test result; M2: 46‐69d post‐positive PCR test result; M3: 78‐107d post‐positive PCR test result; and M4: 106‐155d post‐positive PCR test result.

**TABLE 1:** Participant characteristics

Characteristics	Month 1 (25 ± 5 days)	Month 2 (57 ± 7 days)	Month 3 (87 ± 8 days)	Month 4 (119 ± 13 days)	Month 6 (174 ± 15 days)	Controls
Participants (*n*, M/F)	16 (8 M/8F)	16 (8 M/8F)	12 (7 M/5F)	13 (7 M/6F)	12 (7 M/5F)	20 (5 M/15F)
Age, years	21 ± 1	21 ± 1	22 ± 1	21 ± 1	21 ± 1	23 ± 1^a^
Height, cm	176 ± 10	176 ± 9	176 ± 11	176 ± 10	176 ± 10	167 ± 9
Weight, kg	72 ± 11	73 ± 12	73 ± 7	73 ± 12	74 ± 12	63 ± 7^a^
Body mass index, kg·m^2^	23.5 ± 3.1	23.7 ± 3.09	23.6 ± 2.3	23.7 ± 3.1	23.7 ± 2.9	23 ± 2
Physical activity						
Frequency, days per week	4 ± 1	4 ± 1	4 ± 1	4 ± 1	4 ± 1	4 ± 2
Duration, min per day	41 ± 12	41 ± 12	39 ± 13	40 ± 13	39 ± 13	44 ± 14
Average symptom severity score	4 ± 7	2 ± 6	3 ± 8	1 ± 4	0	—
Female contraceptive use, *n*	6	6	3	4	3	8

*Note*: Mixed‐model repeated‐measures ANOVA (*α* < 0.05; time, 5 levels) was performed to values across month 1 (*n* = 8 M/8F), month 2 (*n* = 8 M/8F), month 3 (*n* = 7 M/5F), month 4 (*n* = 7 M/6F) and month 6 (*n* = 7 M/5F). Data are means ± SD.

^*a*^ *p* < 0.05, group effect.

Participant visit compliance was 78% with 12 of the 16 participants completing at least 5 visits. Two participants (1 M/1F) did not complete the M3‐6 study visits; one other participant (1F) did not complete the M3 study visit; one other participant (1F) did not complete the M4‐6 study visits; one other participant (1F) did not complete M3 or M5 study visits; one other participant (1F) did not complete M5‐6 study visits; three other participants (3 M) did not complete M6 study visit; two other participants (1 M/1F) did not complete M5 study visit. Due to high attrition during M5, the decision was made to collapse the last subject visits from M5 (3M/0F) and M6 (4M/5F) to create a final study visit. The final testing visit (7 M/5F, 174 ± 15d) ranged from 143‐197d post‐positive PCR test result, indicated by “M6” in the results to indicate a final study visit.

### 3.2 SARS‐CoV‐2 symptom severity survey

Most participants (*n* = 15) had mild, lingering symptoms from M1 to M4, while one female participant was consistently asymptomatic from M1 to M6. Symptom severity at each visit for those participants who experienced symptoms (SSS >0) can be found in Table 1. Participants were devoid of any medication usage other than six female subjects using oral contraceptives. Four subjects (3 M/1F) received either one or both doses of the SARS‐CoV‐2 vaccine during the study (three Moderna and one Pfizer). One female participant received her first dose of Moderna between M4 and M6. Two male participants received their first dose between M2 and M3, and the second dose between M4 and M5 (one Moderna and one Pfizer). One other male participant received his first dose of Moderna between M1 and M2, and the second dose between M2 and M3. Vaccinated subjects did not exhibit noticeable differences in measured outcomes compared with the unvaccinated subjects in the current investigation. Further, participants' study visits were scheduled at least 2 weeks following participants' previous vaccine dates to diminish the impact of vaccination effects on outcome variables, a design previously utilized in vaccination studies (Lind et al., 2012). These vaccinated participants' results were generally similar to all other unvaccinated participants' results. Therefore, participants were not separated by vaccination status.

### 3.3 Brachial artery flow‐mediated dilation

**FIGURE 1:**
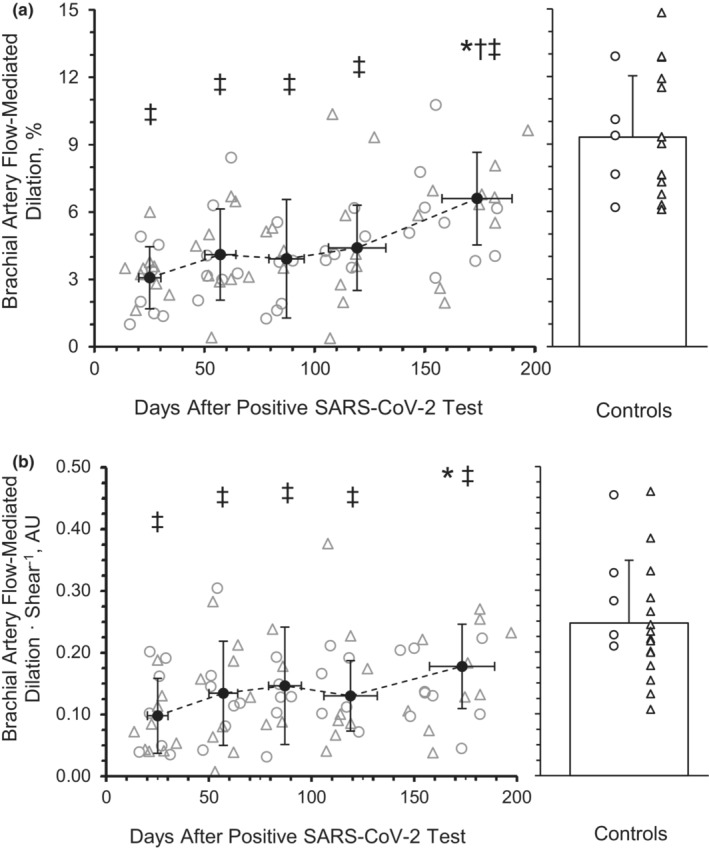
Peak brachial artery flow‐mediated dilation (FMD) (a) and relative to shear (b) during recovery from SARS‐CoV‐2. A mixed‐model repeated‐measures ANOVA (*p* < 0.05; time, 5 levels) was performed to compare peak FMD responses between month 1 (*n* = 8 M/8F), month 2 (*n* = 8 M/8F), month 3 (*n* = 7 M/5F), month 4 (*n* = 7 M/6F), and month 6 (*n* = 7 M/5F). Individual male (circle) and female (triangle) participant data points are denoted. There was a main effect of time *p* < 0.001, with month 6 being significantly higher than month 1 (*p* < 0.001). *p < 0.05 compared with month 1. ^†^ *p* < 0.05 compared with month 2. ^‡^ *p* < 0.05, group effect. Data are means ± SD.

Measurements of brachial artery FMD are presented in Table 2. There were no main effects of time for baseline brachial artery diameter (*p* = 0.428, *η* ^2^ = 0.060), time to peak vasodilation (*p* = 0.853, *η* ^2^ = 0.031), absolute change in brachial artery diameter from baseline to peak vasodilation (*p* = 0.178, *η* ^2^ = 0.109), peak dilation (*p* = 0.658, *η* ^2^ = 0.036), sum of shear at peak dilation (*p* = 0.230, *η* ^2^ = 0.023), supine SBP (*p* = 0.075, *η* ^2^ = 0.144), supine DBP (*p* = 0.307, *η* ^2^ = 0.0567), supine MAP (*p* = 0.112, *η* ^2^ = 0.116), peak velocity (*p* = 0.522, *η* ^2^ = 0.092), and peak velocity relative to MAP (*p* = 0.999, *η* ^2^ = 0.075) across time. There was a main effect of time for %FMD at peak diameter (Figure 1a; *p* < 0.001, *η* ^2^ = 0.259). The %FMD at peak diameter was not different from M1 (3.06 ± 1.39%) to M2 (4.10 ± 2.03%, *p* = 0.453, Hedge's *g* = −0.509), M3 (3.91 ± 2.64%, *p* = 0.850, Hedge's *g* = −0.417) or M4 (4.40 ± 1.90%, *p* = 0.228, Hedge's *g* = −0.656) but was significantly greater at M6 (6.60 ± 2.07%, *p* < 0.001, Hedge's *g* = −1.737). There was a main group effect between the SARS‐CoV‐2 group and the healthy retrospective control group for %FMD (*p* < 0.001). The %FMD at peak diameter was lower among participants with SARS‐CoV‐2 at M1 (*p* < 0.001, Hedge's *g* = 2.816), M2 (*p* < 0.001, Hedge's *g* = 2.349), M3 (*p* < 0.001, Hedge's *g* = 2.434), M4 (*p* < 0.001, Hedge's *g* = 2.215), and M6 (*p* = 0.001, Hedge's *g* = 1.223) compared to the healthy retrospective control group (9.30 ± 2.73). Additionally, %FMD at peak diameter at M2 (*p* = 0.018) but not M3 (*p* = 0.054) or M4 (*p* = 0.056), was significantly different from M6. There was a main effect of time for %FMD relative to shear (Figure 1b; *p* = 0.039, *η* ^2^ = 0.151). The %FMD relative to shear was not different from M1 (0.10 ± 0.06 AU) at M2 (0.13 ± 0.08 AU; *p* = 0.631, Hedge's *g* = −0.484), M3 (0.15 ± 0.09 AU; *p* = 0.539, Hedge's *g* = −0.644), or M4 (0.13 ± 0.06 AU; *p* = 0.587, Hedge's *g* = −0.429) but was significantly greater at M6 (0.18 ± 0.07 AU; *p* = 0.017, Hedge's *g* = 1.065). There was a main group effect between the SARS‐CoV‐2 group and the healthy retrospective control group for %FMD relative to shear (*p* < 0.001, *η* ^2^ = 0.308). The %FMD at peak diameter relative to shear was lower among participants with SARS‐CoV‐2 at M1 (*p* < 0.001, Hedge's *g* = 1.827), M2 (*p* < 0.001, Hedge's *g* = 1.384), M3 (*p* = 0.001, Hedge's *g* = 1.238), M4 (*p* < 0.001, Hedge's *g* = 1.434), and M6 (*p* = 0.021, Hedge's *g* = 0.852) compared to the healthy retrospective control group (0.25 ± 0.01).

**TABLE 2:** Flow‐mediated dilation

	Month 1 (25 ± 5 days)	Month 2 (57 ± 7 days)	Month 3 (87 ± 8 days)	Month 4 (119 ± 13 days)	Month 6 (174 ± 15 days)	Controls
Participants (*n*, M/F)	16 (8 M/8F)	16 (8 M/8F)	12 (7 M/5F)	13 (7 M/6F)	12 (7 M/5F)	20 (5 M/15F)
Baseline diameter, mm	4.22 ± 0.63	4.08 ± 0.56	4.13 ± 0.56	4.23 ± 0.57	3.84 ± 0.54	3.67 ± 0.49
Peak diameter, mm	4.35 ± 0.63	4.25 ± 0.59	4.29 ± 0.52	4.40 ± 0.58	4.07 ± 0.58	3.98 ± 0.53
Δ Diameter, mm	0.13 ± 0.07	0.17 ± 0.09	0.15 ± 0.09	0.18 ± 0.06	0.23 ± 0.14	0.31 ± 0.11
Time to Peak, sec	53 ± 23	50 ± 30	46 ± 29	55 ± 23	47 ± 21	58 ± 23
Sum shear value at peak, AU	36,806 ± 16,436	38,969 ± 20,733	28,017 ± 13,711	37,195 ± 15,377	42,457 ± 19,657	42,404 ± 17,636
Supine systolic blood pressure, mmHg	129 ± 15	121 ± 9	117 ± 6	119 ± 14	118 ± 9	112 ± 13
Supine diastolic blood pressure, mmHg	73 ± 7	71 ± 8	69 ± 7	71 ± 11	68 ± 8	78 ± 8
Supine mean arterial pressure, mmHg	92 ± 9	88 ± 8	85 ± 6	87 ± 47	84 ± 8	89 ± 6
Resting Brachial Artery BF, ml·min^−1^	97 ± 43	89 ± 42	75 ± 30	87 ± 47	77 ± 39	67 ± 26
Resting Brachial Artery VC, ml·min^−1^·m^−1^	1.05 ± 0.47	1.02 ± 0.51	0.90 ± 0.39	1.03 ± 0.62	0.93 ± 0.48	0.74 ± 0.26
Reactive Hyperemia, AUC	387 ± 133	393 ± 192	340 ± 111	416 ± 161	391 ± 187	317 ± 117
Reactive Hyperemia/MAP, AUC·mmHg^−1^	4.26 ± 1.59	4.50 ± 2.24	4.06 ± 1.42	4.87 ± 1.99	4.73 ± 2.39	3.57 ± 1.29
Peak Velocity, cm·s^−1^	50.5 ± 10.8	55.9 ± 19.1	44.1 ± 7.7	48.4 ± 9.6	52.8 ± 12.4	53.7 ± 13.0
Peak Velocity/MAP, cm·s^−1^·mmHg^−1^	0.56 ± 0.15	0.64 ± 0.22	0.53 ± 0.12	0.56 ± 0.12	0.64 ± 0.19	0.61 ± 0.15

*Note*: Mixed‐model repeated‐measures ANOVA (*α* < 0.05; time, 5 levels) was performed to compare values across month 1 (*n* = 8 M/8F), month 2 (*n* = 8 M/8F), month 3 (*n* = 7 M/5F), month 4 (*n* = 7 M/6F) and month 6 (*n* = 7 M/5F). Δ diameter: change in vessel diameter from baseline to peak, FMD/Shear: flow‐mediated dilation response related to shear stimulus, Data are means ± SD.

Abbreviations: BF, blood flow; VC, vascular conductance.

### 3.4 Reactive hyperemia

**FIGURE 2:**
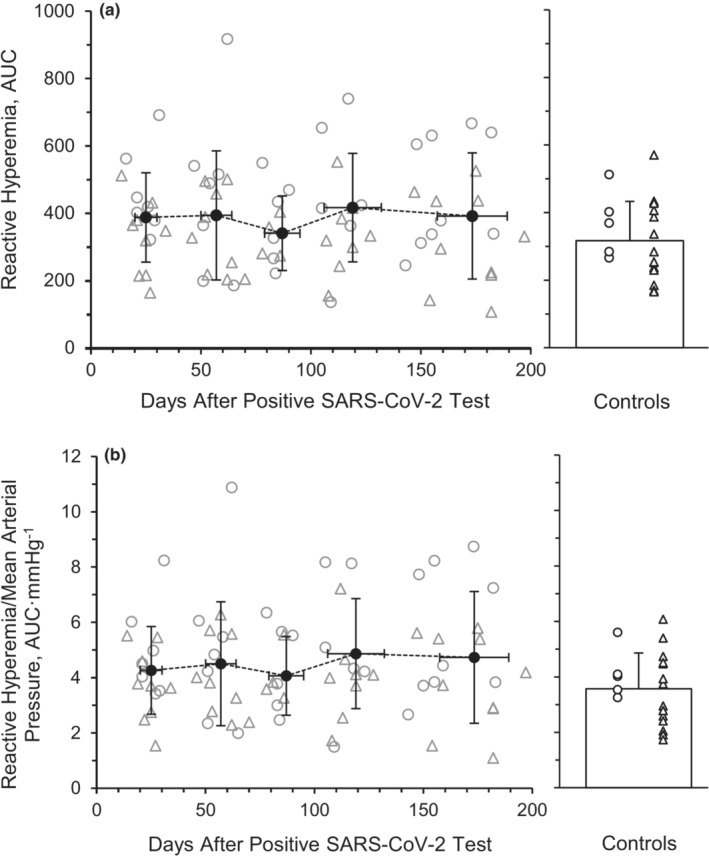
Brachial artery reactive hyperemia expressed as area under the curve (AUC) (a) and made relative to mean arterial pressure (AUC·mmHg^−1^) (b) during recovery from SARS‐CoV‐2. A repeated‐measures ANOVA (*p* < 0.05; time, 5 levels) was performed to compare RH responses to a 5‐min cuff occlusion between month 1 (*n* = 8 M/8F), month 2 (*n* = 8 M/8F), month 3 (*n* = 7 M/5F), month 4 (*n* = 7 M/6F), and month 6 (*n* = 7 M/5F). Individual male (circle) and female (triangle) participant data points are denoted. Data are means ± SD.

Brachial artery RH results are displayed in Table 2. Resting brachial artery BF and VC was not different across the 6‐month period (*p* = 0.560, *η* ^2^ = 0.038 and *p* = 0.882, *η* ^2^ = 0.014, respectively). Additionally, RH, assessed by AUC (Figure 2a), and made relative to mean arterial pressure (RH/MAP, Figure 2b) was not different across the 6 months (*p* = 0.665, *η* ^2^ = 0.023 and *p* = 0.781, *η* ^2^ = 0.022, respectively). There was not a main effect for RH (*p* = 0.420, *η* ^2^ = 0.057) and RH/MAP (*p* = 0.370, *η* ^2^ = 0.062) between the SARS‐CoV‐2 group and the healthy retrospective control group.

### 3.5 Femoral artery single passive limb movement

**FIGURE 3:**
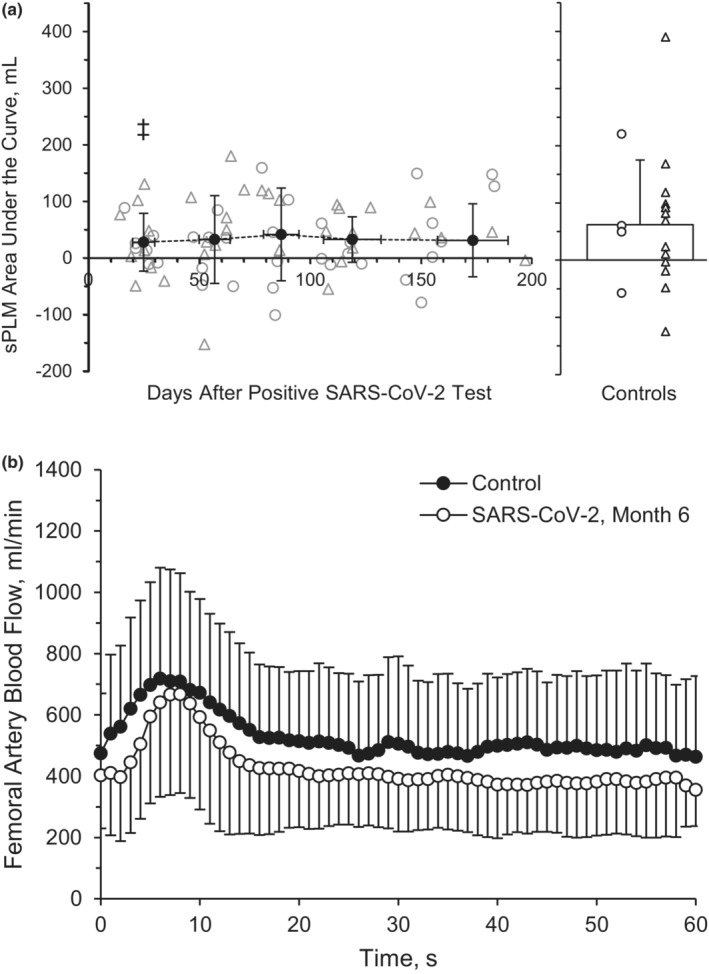
Single passive limb movement. Common femoral artery blood flow, represented by area under the curve (AUC) at 60 s during recovery from SARS‐CoV‐2 and retrospective healthy control participants (a). A repeated‐measures ANOVA (*p* < 0.05; time, 5 levels) was performed to compare BF responses between month 1 (*n* = 8 M/8F), month 2 (*n* = 8 M/8F), month 3 (*n* = 7 M/5F), month 4 (*n* = 7 M/6F), and month 6 (*n* = 7 M/5F). Femoral artery blood flow during month 6 for participants with SARS‐CoV‐2 and retrospective health control participants (b). Two‐tailed student's *t*‐tests for two samples of equal variance were performed between control (*n* = 5 M/15F) and SARS‐CoV‐2 at month 6 (*n* = 7 M/5F) participants. Individual male (circle) and female (triangle) participant data points are denoted (a). ^‡^ *p* < 0.05, group effect. Data are means ± SD.

Measurements of common femoral artery sPLM are displayed in Table 3. Baseline femoral artery BF (*p* = 0.808), peak BF during the sPLM (*p* = 0.829), ΔPeak_BF_ (*p* = 0.938), and AUC60_BF_ (Figure 3a, *p* = 0.991) were not different across the six‐month recovery period during the sPLM. Baseline VC (*p* = 0.405), peak VC (*p* = 0.780), ΔPeak_VC_ (*p* = 0.795), and AUC60_VC_ (*p* = 0.473) were also not different across time during the sPLM. There was not a main group effect between the SARS‐CoV‐2 group and the healthy retrospective control group for baseline BF (*p* = 0.652, *η* ^2^ = 0.032), peak BF during the sPLM (*p* = 0.736, *η* ^2^ = 0.024), or ΔPeak_BF_ (*p* = 0.861, *η* ^2^ = 0.020; Figure 3b). However, there was a main group effect for AUC60_BF_ (*p* = 0.047, *η* ^2^ = 0.177). The AUC60_BF_ was different between groups at M1 (*p* = 0.034, Hedge's *g* = 0.716) but not at M2 (*p* = 0.054, Hedge's *g* = 0.649), M3 (*p* = 0.149, Hedge's *g* = 0.527), M4 (*p* = 0.069, Hedge's *g* = 0.650), or M6 (*p* = 0.067, Hedge's *g* = 0.671) compare to the healthy retrospective control group (Table 3).

**TABLE 3:** Passive limb movement

	Month 1 (25 ± 5 days)	Month 2 (57 ± 7 days)	Month 3 (87 ± 8 days)	Month 4 (119 ± 13 days)	Month 6 (174 ± 15 days)	Controls
Participants (*n*, M/F)	16 (8 M/8F)	16 (8 M/8F)	12 (7 M/5F)	13 (7 M/6F)	12 (7 M/5F)	20 (5 M/15F)
Blood flow, sPLM						
Baseline, ml⋅min^−1^	416 ± 207	384 ± 118	455 ± 244	346 ± 245	403 ± 174	475 ± 196
Peak, ml⋅min^−1^	680 ± 313	631 ± 220	775 ± 413	640 ± 379	704 ± 336	719 ± 351
Δ peak, ml⋅min^−1^	263 ± 185	248 ± 187	320 ± 207	295 ± 177	301 ± 214	244 ± 205
AUC 60, ml	27.6 ± 51.0^b^	32.5 ± 77.5	41.4 ± 82.0	32.5 ± 40.2	30.9 ± 64.6	62 ± 113
Vascular Conductance, sPLM						
Baseline, ml⋅mmHg⋅min^−1^	5.45 ± 3.35	4.24 ± 1.52	4.68 ± 2.02	3.55 ± 2.27	4.78 ± 1.92	—
peak, ml⋅mmHg⋅min^−1^	9.16 ± 4.97	8.75 ± 5.70	8.13 ± 3.28	7.37 ± 4.32	9.11 ± 3.04	—
Δ peak, ml⋅mmHg⋅min^−1^	3.71 ± 2.69	4.52 ± 4.67	3.45 ± 1.66	3.82 ± 2.29	4.33 ± 2.10	—
AUC 60, ml	0.28 ± 0.77	0.51 ± 0.95	0.41 ± 0.78	0.51 ± 0.83	0.59 ± 1.06	—
Blood Flow, cPLM						
Baseline, ml⋅min^−1^	392 ± 160	376 ± 122	427 ± 187	360 ± 235	433 ± 179	481 ± 199
Peak, ml⋅min^−1^	804 ± 267	807 ± 287	909 ± 354	957 ± 563	977 ± 416	942 ± 394
Δ peak, ml⋅min^−1^	412 ± 129	431 ± 204	482 ± 305	596 ± 397	544 ± 318	462 ± 291
AUC 60, ml	171.3 ± 68.4	209.0 ± 104.0	219.9 ± 162.5	240.4 ± 171.5	220.2 ± 120.6	183 ± 166
Vascular conductance, cPLM						
Baseline, ml⋅mmHg⋅min^−1^	4.57 ± 1.67	4.17 ± 1.07	4.68 ± 2.03	3.82 ± 2.10	4.79 ± 1.93	—
Peak, ml⋅mmHg^−1^⋅min^−1^	9.81 ± 2.70	11.27 ± 5.87	9.95 ± 4.03	11.50 ± 7.23	10.59 ± 4.56	—
Δ peak, ml⋅mmHg^−1^⋅min^−1^	5.24 ± 1.61	7.10 ± 5.31	5.27 ± 3.10	7.68 ± 5.41	5.80 ± 3.74	—
AUC 60, ml⋅mmHg^−1^	2.24 ± 1.06	2.32 ± 1.2	2.10 ± 1.48	2.96 ± 2.30	2.04 ± 1.48	—
Cardiac output, cPLM						
Baseline, L⋅min^−1^	6.39 ± 1.05	5.29 ± 1.58	5.34 ± 1.62	5.40 ± 1.19	6.46 ± 3.12	—
Peak, L⋅min^−1^	8.37 ± 3.19	6.98 ± 3.04	6.12 ± 1.83	6.70 ± 1.70	7.65 ± 3.32	—
Δ peak, L⋅min^−1^	1.98 ± 2.69	1.69 ± 2.24	0.78 ± 0.40	1.30 ± 0.62	1.19 ± 0.64	—
AUC 60, L	0.64 ± 1.45	0.68 ± 0.58	0.39 ± 0.37	0.80 ± 0.53	0.46 ± 0.40	—
Stroke volume, cPLM						
Baseline, ml	95.44 ± 16.56	86.71 ± 27.53	83.55 ± 24.32	85.12 ± 17.75	93.40 ± 20.26	—
peak, ml	99.68 ± 19.67	95.54 ± 26.27	92.28 ± 24.75	96.01 ± 20.49	103.02 ± 24.40	—
Δ peak, ml	4.24 ± 8.24	8.83 ± 7.87	8.73 ± 9.09	10.89 ± 5.34	9.62 ± 7.61	—
AUC 60, ml	−2.15 ± 11.52	2.92 ± 7.52	3.84 ± 8.03	5.80 ± 3.96*	1.51 ± 3.60	—
Mean arterial pressure, cPLM						
Baseline, mmHg	89.43 ± 6.69	92.77 ± 10.34	91.87 ± 7.86	84.74 ± 8.59	84.72 ± 13.01	—
Peak, mmHg	96.27 ± 12.04	101.77 ± 12.02	102.44 ± 7.09	92.97 ± 14.66	94.66 ± 15.25	—
Δ peak, mmHg	6.84 ± 10.35	9.00 ± 6.07	10.57 ± 5.78	8.24 ± 10.57	9.94 ± 5.75	—
AUC 60, mmHg^a^	0.70 ± 11.15	3.40 ± 5.10	6.00 ± 6.05	2.98 ± 8.80*	4.84 ± 5.26	—
Heart rate, cPLM						
Baseline, BPM	67.80 ± 10.8	61.69 ± 8.39	63.97 ± 7.90	63.29 ± 10.51	66.27 ± 18.97	—
Peak, BPM	89.52 ± 33.16	76.81 ± 19.22	72.62 ± 10.24	72.11 ± 11.29	75.55 ± 20.48	—
Δ peak, BPM	21.71 ± 25.73	15.12 ± 21.74	8.65 ± 3.80	8.82 ± 3.57	9.28 ± 4.98	—
AUC 60, BPM	9.74 ± 10.02	5.70 ± 6.80	3.74 ± 2.42	5.00 ± 3.36	4.40 ± 3.07	—

*Note*: Mixed‐model repeated‐measures ANOVA (*α* < 0.05; time, 5 levels) was performed to compare values across month 1 (*n* = 8 M/8F), month 2 (*n* = 8 M/8F), month 3 (*n* = 7 M/5F), month 4 (*n* = 7 M/6F) and month 6 (*n* = 7 M/5F). Data are means ± SD.

Abbreviations: AUC 60, area under the curve at 60‐seconds; cPLM, continuous passive limb movement; sPLM, single passive limb movement; Δ peak, change from baseline to peak.

^a^ Main effect of time, *p* < 0.05.

^b^ *p* < 0.05, group effect.

### 3.6 Femoral artery continuous passive limb movement

**FIGURE 4:**
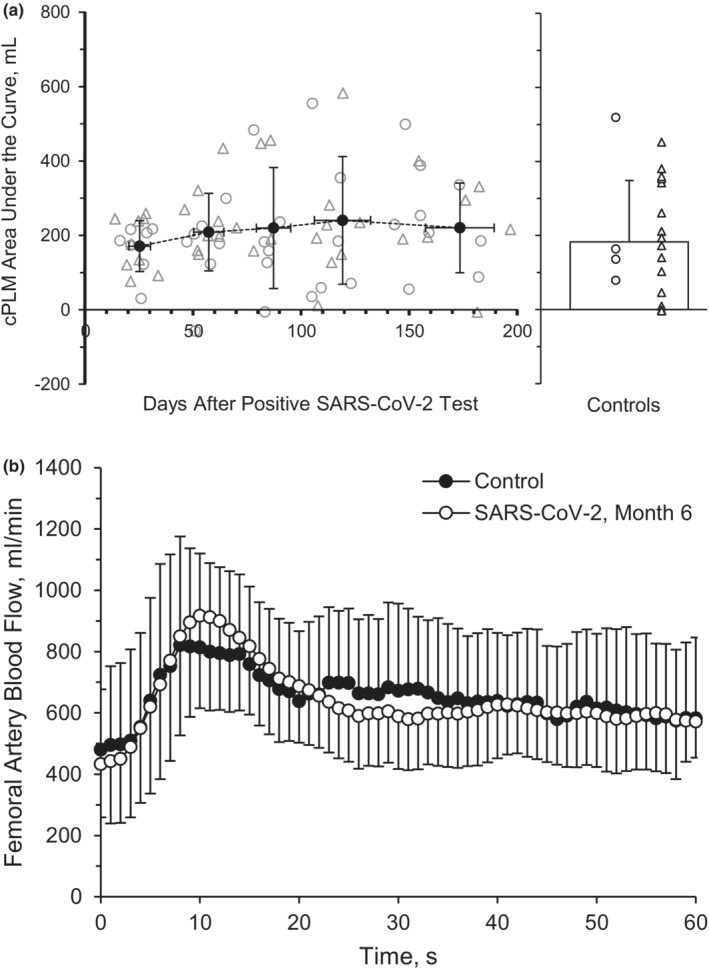
Continuous passive limb movement. Common femoral artery blood flow, represented by area under the curve (AUC) at 60 s (a) and vascular conductance at 60 s, represented by AUC (b) during recovery from SARS‐CoV‐2. A repeated‐measures ANOVA (*p* < 0.05; time, 5 levels) was performed to compare BF responses between month 1 (*n* = 8 M/8F), month 2 (*n* = 8 M/8F), month 3 (*n* = 7 M/5F), month 4 (*n* = 7 M/6F) and month 6 (*n* = 7 M/5F). Individual male (circle) and female (triangle) participant data points are denoted (a). Data are means ± SD.

Measurements of femoral artery cPLM are displayed in Table 3. Baseline femoral artery BF (*p* = 0.817, *η* ^2^ = 0.055), peak BF (*p* = 0.604, *η* ^2^ = 0.034), ΔPeak_BF_ (*p* = 0.379, *η* ^2^ = 0.051), and AUC60_BF_ (Figure 4a, *p* = 0.424, *η* ^2^ = 0.031), were not different across time during the cPLM. Baseline VC (*p* = 0.659), peak VC (*p* = 0.845), ΔPeak_VC_ (*p* = 0.420), and AUC60_VC_ (*p* = 0.815) were also not different across time during the cPLM. There was not a main group effect between the SARS‐CoV‐2 group and the healthy retrospective control group for baseline BF (*p* = 0.451, *η* ^2^ = 0.038), peak BF during the cPLM (*p* = 0.717, *η* ^2^ = 0.030), ΔPeak_BF_ (*p* = 0.503, *η* ^2^ = 0.020), or AUC60_BF_ (*p* = 0.752, *η* ^2^ = 0.052; Figure 4b).

### 3.7 Central hemodynamic response to cPLM

Central hemodynamic measurements during the cPLM are displayed in Table 3. The baseline CO (*p* = 0.074), peak CO response (*p* = 0.163), AUC60_CO_ (*p* = 0.209), and ΔPeak_CO_ (*p* = 0.056) were not different across time. The MAP at baseline (p = 0.107), peak (*p* = 0.135), ΔPeak_MAP_ (*p* = 0.785), and AUC60 (*p* = 0.683) was not different across time. The HR at baseline (*p* = 0.389), peak (*p* = 0.326), ΔPeak_HR_ (*p* = 0.393), and AUC60_HR_ (*p* = 0.248) were not different across time. The SV at baseline (*p* = 0.423), peak (*p* = 0.828), and ΔPeak_SV_ (*p* = 0.179) were not different across time. After the removal of outliers, there was a main effect of time (*p* = 0.007) for AUC60_SV_. There were no differences from M1 to M2 (*p* = 0.103), M3 (*p* = 0.716), or M6 (*p* = 0.977) but AUC60_SV_ was significantly greater at M4 compared with M1 (*p* = 0.008).

### 3.8 Markers of oxidative stress, inflammation, nitric oxide bioavailability, and antioxidant levels

Measurements of oxidative stress, inflammation, nitric oxide (NO) bioavailability, and antioxidant levels are presented in Table 4. Due to the amount of adequate blood samples available, 8–14 participant samples were available for each biomarker, as indicated in Table 4. Blood samples were analyzed for every study visit for all biomarkers, except protein carbonyl, which was only analyzed for M1, M3, and M6 study visits. There was no main effect of time for protein carbonyl (*p* = 0.329) across time. Additionally, there was no main effect for time for TBARS (*p* = 0.424), IL‐6 (*p* = 0.207), CRP (*p* = 0.368), nitrite (*p* = 0.942), and SOD (*p* = 0.334) across time. The individual participant's slopes (7 M/5F) from M1 to M6 for %FMD were not associated with the individual participant slopes (7 M/5F) from M1 to M6 for CRP (*R* = 0.024, *p* = 0.940), IL‐6 (*R* = 0.124, *p* = 0.686), SOD (*R* = 0.186, *p* = 0.526), TBARS (*R* = −0.375, *p* = 0.186), protein carbonyl (*R* = −0.046, *p* = 0.881), or nitrite (*R* = −0.445, *p* = 0.111). The slope from M1 to M6 for %FMD/Shear was not associated with slope from M1 to M6 for CRP (*R* = 0.419, *p* = 0.175), IL‐6 (*R* = 0.163, *p* = 0.596), SOD (*R* = −0.170, *p* = 0.561), TBARS (*R* = −0.228, *p* = 0.433), protein carbonyl (*R* = −0.171, *p* = 0.577), or nitrite (*R* = 0.013, *p* = 0.965). The slope from M1 to M6 for sPLM BF AUC was not associated with slope from M1 to M6 CRP (*R* = 0.360, *p* = 0.250), SOD (*R* = 0.327, *p* = 0.254), TBARS (*R* = 0.001, *p* = 0.997), protein carbonyl (*R* = −0.126, *p* = 0.681), or nitrite (*R* = 0.139, *p* = 0.636), although there was a positive association with IL‐6 (*R* = 0.665, *p* = 0.013). The slope from M1 to M6 for cPLM BF AUC was not associated with slope from M1 to M6 for CRP (*R* = 0.425, *p* = 0.168), IL‐6 (*R* = 0.453, *p* = 0.120), SOD (*R* = 0.494, *p* = 0.073), TBARS (*R* = 0.027, *p* = 0.927), protein carbonyl (*R* = −0.169, *p* = 0.581), or nitrite (*R* = 0.004, *p* = 0.989).

**TABLE 4:** Blood analysis

	Month 1 (25 ± 5 days)	Month 2 (57 ± 7 days)	Month 3 (87 ± 8 days)	Month 4 (119 ± 13 days)	Month 6 (174 ± 15 days)
Participants (*n*, M/F)	16 (8 M/8F)	16 (8 M/8F)	12 (7 M/5F)	13 (7 M/6F)	12 (7 M/5F)
Oxidative stress					
TBARS, μM	19.8 ± 5.4, *n* = 14 (8 M/6F)	19.6 ± 5.7, *n* = 14 (8 M/6F)	19.6 ± 4.7, *n* = 12 (7 M/5F)	20.3 ± 3.3, *n* = 11 (6 M/5F)	18.0 ± 5.3, *n* = 11 (7 M/4F)
Protein Carbonyl, nmol⋅mg^−1^	37.9 ± 21.0, *n* = 12 (6 M/6F)	—	52.4 ± 42.7, *n* = 11 (6 M/5F)	—	31.4 ± 8.6, *n* = 10 (6 M/4F)
Inflammation					
Interleukin‐6, pg⋅ml^−1^	30.3 ± 26.7, *n* = 12 (7 M/5F)	30.1 ± 29.0, *n* = 12 (7 M/5F)	26.4 ± 22.3, *n* = 11 (7 M/4F)	26.7 ± 21.5, *n* = 11 (7 M/4F)	23.1 ± 21.8, *n* = 11 (7 M/4F)
C‐Reactive Protein, pg⋅mL^−1^	154.2 ± 171.4, *n* = 12 (7 M/5F)	87.3 ± 88.0, *n* = 10 (5 M/5F)	137.6 ± 120.6, *n* = 10 (6 M/4F)	86.2 ± 101.0, *n* = 9 (5 M/4F)	155.5 ± 196.9, *n* = 8 (5 M/3F)
Antioxidants					
Superoxide dismutase, U⋅ml^−1^	31.9 ± 2.8, *n* = 14 (8 M/6F)	30.8 ± 4.6, *n* = 14 (8 M/6F)	31.3 ± 3.2, *n* = 12 (7 M/5F)	32.0 ± 4.2, *n* = 12 (7 M/5F)	30.0 ± 2.2, *n* = 11 (7 M/4F)
Nitric oxide precursor					
Nitrite, μmol⋅L^−1^	37.5 ± 12.0 *n* = 12 (7 M/5F)	34.1 ± 14.5, *n* = 13 (7 M/6F)	37.5 ± 10.6, *n* = 11 (6 M/5F)	35.5 ± 13.0, *n* = 11 (7 M/4F)	34.9 ± 11.2, *n* = 11 (7 M/4F)

*Note*: Mixed‐model repeated‐measures ANOVA (*α* < 0.05; time, 5 levels) was performed to values across month 1 (*n* = 8 M/8F), month 2 (*n* = 8 M/8F), month 3 (*n* = 7 M/5F), month 4 (*n* = 7 M/6F) and month 6 (*n* = 7 M/5F). Data are means ± SD.

## 4 DISCUSSION

The purpose of this study was to determine the natural time course for potential recovery from acute vascular dysfunction by tracking vascular function in the arms and legs, as well as various biomarkers, for a 6‐month period post‐SARS‐CoV‐2 infection. In support of our hypothesis, %FMD increased throughout the 6‐month period, and was significantly greater at the six‐month time point compared with the study entry, indicating improvements in conduit vascular function in the brachial artery. Brachial artery RH, a measure of microvascular function, was unaltered 6 months following SARS‐CoV‐2 infection. Likewise, the hyperemic response to the mobility muscle microvascular assessments sPLM and cPLM were unaltered 6 months following SARS‐CoV‐2 infection, despite a lower sPLM AUC60_BF_ for young adults recovering from SARS‐CoV‐2 compared to healthy retrospective control subjects acutely after infection. Interestingly, there were no differences in circulating biomarker levels throughout the 6‐month testing period, nor did the slope over the 6‐month period for the circulating biomarkers correlate with the functional vascular biomarkers. However, the results from the FMD and sPLM measurements provide evidence for impaired vascular function following SARS‐CoV‐2 infection. Together, these data suggest persistent consequences to cardiovascular health among those recovering from SARS‐CoV‐2, even with mild illness and in young, otherwise healthy adults.

### 4.1 Brachial artery flow‐mediated dilation

Brachial artery FMD is an assessment of macrovascular function (Thijssen et al., 2011) which correlates with coronary artery vascular function and is predictive of cardiovascular disease risk (Broxterman et al., 2019). SARS‐CoV‐2 is known to induce a systemic inflammatory response (Paybast et al., 2020). Previous studies demonstrate there is also a direct inflammatory response in endothelial cells (Varga et al., 2020), which could lead to vascular dysfunction and directly impact BF regulation (Bhagat & Vallance, 1997; Hingorani et al., 2000; Zimmer et al., 2011). From our initial findings (Ratchford et al., 2020), the acute decrease in %FMD among young adults with SARS‐CoV‐2 (2.7%) cross‐sectionally compared to healthy young adults (8.8%) is concerning, as this measurement is associated with a higher risk for cardiovascular events, such as heart attack, stroke, or death (Yeboah et al., 2009). As mentioned, other groups have attempted to provide evidence of the potentially long‐lasting effects of SARS‐CoV‐2 on vascular function among hospitalized older adults (Ambrosino et al., 2021) which may be related to disease severity (Riou et al., 2021) and last for several months (Lambadiari et al., 2021). Similar to the aforementioned studies in older adults, an additional investigation in otherwise healthy young adults recovering from SARS‐CoV‐2 tested at a single time point, between 1 and 5 months after infection, providing further evidence of a symptom‐dependent, negative association with vascular function as indicated by %FMD (Nandadeva et al., 2021). However, this study did not investigate when this vascular improvement occurs during recovery, with measurements being taken once per participant, and at various time points across 4–21 weeks post‐infection. This current investigation sought to bridge this gap and provide longitudinal data on younger individuals with mild or no symptoms. We provide measurements at multiple time points to indicate %FMD at peak dilation improved in young adults by 6 months after SARS‐CoV‐2 infection which remains lower than healthy controls in the current investigation (Figure 1). Similar findings among cross‐sectional investigations have revealed these decrements in macrovascular function may persist for at least 12 months following SARS‐CoV‐2 infection (Faria et al., 2022; Ikonomidis et al., 2022; Zota et al., 2022), suggesting long‐term vascular decrements.

SARS‐CoV‐2 elicits the cytokine storm which can cause an inflammatory state (Paybast et al., 2020) and elevate resting sympathetic levels (Stute, Stickford, Province, et al., 2021). Our group also investigated the effects of SARS‐CoV‐2 on the autonomic function in young healthy individuals during the acute phase of infection (Stute, Stickford, Province, et al., 2021). These results indicate mildly higher sympathetic activity in those infected, compared with a healthy control group. Previous studies show that an elevated sympathetic drive can impede the FMD response (Hijmering et al., 2002; Thijssen et al., 2006). Likewise, a lower heart‐rate‐variability in individuals with a SARS‐CoV‐2 infection, but displaying mild symptoms, compared with healthy, control counterparts (Kurtoglu et al., 2022; Solinski et al., 2022). Furthermore, persistent sympathetic overactivation (66% higher MSNA) along with blunted vascular function (45% lower FMD) have been observed cross‐sectionally among older adults who were hospitalized with COVID‐19 and were studied 1 year after infection compared to well‐matched control subjects (Faria et al., 2022), further suggesting long‐lasting autonomic dysfunction among those previously infected with COVID‐19. With these studies in mind, the initial reduction in %FMD may be attributed to autonomic dysregulation. However, the autonomic function should be investigated in long‐term recovery in individuals similar to this participant pool to further support that sympathetic activity is in fact impeding the FMD response.

### 4.2 Reactive hyperemia

RH is used as a measurement of microvascular function. Unlike FMD, RH response to cuff occlusion and release is minimally influenced by NO (Engelke et al., 1996). The lower %FMD and normal RH response observed acutely (Ratchford et al., 2020) along with the increases seen with %FMD and lack of change in RH after several months following infection in our study may indicate SARS‐CoV‐2 may preferentially affect NO bioavailability and macrovascular function more than microvascular function among these young mild cases of SARS‐CoV‐2. In our previous study, the group of young adults 3 to 4 weeks after contracting SARS‐CoV‐2 had similar RH values compared with the healthy controls (Ratchford et al., 2020). Tehrani et al. found that severe SARS‐CoV‐2 patients who required mechanical ventilation displayed impaired microvascular function and particularly lower endothelial function, which persisted for 3 months post‐infection (Tehrani & Gille‐Johnson, 2021), indicating long‐term microvascular decrements in severe cases. The previous investigation by Nandadeva et al. observed a symptom‐dependent association with microvascular function in young adults with SARS‐CoV‐2 as well (Nandadeva et al., 2021), with a lack of difference between the COVID‐19 group and the control group. However, when divided into COVID‐19 subgroups based on symptom presence, the symptomatic group had lower RH values compared with the asymptomatic and control groups, while the asymptomatic participants' FMD values did not differ from the control group. The lack of change in RH observed in the current investigation, nor any cross‐sectional difference at Month 6 compared to healthy controls (Figure 2), could be attributed to the participant population displaying mild symptoms and may represent a divergence in vascular dysfunction and recovery throughout the vascular tree.

### 4.3 Femoral artery single passive limb movement

PLM is a novel, non‐invasive assessment of mobility muscle microvascular function and has been observed to be greater than 80% dependent on NO bioavailability (Gifford & Richardson, 2017; Trinity et al., 2010). Those with decreased cardiovascular health, such as elderly individuals and those with heart failure tend to have a blunted PLM‐induced hyperemic response (Francisco et al., 2021; Gifford & Richardson, 2017). The use of the sPLM, as opposed to the cPLM, evokes peripheral hemodynamic responses while minimizing central hemodynamic responses, provoking NO‐dependent microvascular vasodilation (Ives et al., 2016; Venturelli et al., 2017). Our recent observation, utilizing a preliminary cohort of subjects, of an acute decline in the hyperemic response to sPLM among young adults 3–4 weeks after SARS‐CoV‐2 infection compared with healthy controls (Ratchford et al., 2020), indicates a reduced ability of the small arterioles to dilate when provoked by physical distortion. These results align with those observed in the elderly 2 to 4 months after hospitalization for SARS‐CoV‐2 infection (Paneroni et al., 2021). At rest, the elderly cohort displayed similar femoral artery diameter, but a significantly lower blood velocity and femoral BF (~100 ml/min) compared with a healthy control group. Additionally, an ~150 ml/min lower peak femoral BF, ~125 ml/min lower ΔBFpeak and lower AUC was observed in the COVID group compared with control after the sPLM movement (Paneroni et al., 2021).

Interestingly, the current investigation observed no improvements in the hyperemic response of the sPLM over the course of 6 months. Differential responses in vascular function and structure between the arms and the legs, during both rest and exercise, have been noted by our group (Augenreich et al., 2020; Ratchford et al., 2020) and others (Newcomer et al., 2004, 2005; Parker et al., 2006; Richardson et al., 2006). Further, there is growing evidence that FMD is not primarily NO‐mediated (Green et al., 2014; Wray et al., 2013), which could represent divergent mechanisms between the findings of improved FMD and a lack of change in either sPLM or NO in these 6 months following SARS‐CoV‐2 infection. Given the similar leg BF responses between participants with SARS‐CoV‐2 6 months after infection and healthy controls to sPLM (Figure 3) and cPLM (Figure 4), perhaps limb‐dependent (arm versus leg) and/or other cellular mechanisms (NO versus non‐NO‐mediated vasodilation) may be responsible for these limb‐specific and macro‐ versus microvascular findings. Yet, more work is certainly needed to further examine the vascular recovery rates across the upper and lower limbs among those recovering from SARS‐CoV‐2, quite possibly using additional methodologies to discern NO and non‐NO‐mediated dilatory mechanisms.

### 4.4 Femoral artery continuous passive limb movement

SARS‐CoV‐2 infection can cause an increase in mitochondrial reactive oxygen species (ROS), evoking increases in oxidative stress and inflammation as well as decreases in bioavailability of the potent endothelial‐derived vasodilator, NO (Fedorova et al., 2014; Morris et al., 2020; Pearce et al., 2020; Saeed & Mancia, 2021). Endothelial‐derived NO production has been noted to play a crucial role in the change in vascular tone during PLM (Gifford & Richardson, 2017). The movement of the leg elicits endothelial NO release and other dilatory mechanisms, resulting in rapid dilation of the vascular bed. The vascular decrements caused by SARS‐CoV‐2 may contribute to blunted exercising BF (Stute, Stickford, Stickford, et al., 2021) and whole‐body aerobic capacity (Acar et al., 2021), ultimately impacting quality of life among SARS‐CoV‐2 survivors. The current investigation does not show significant improvements in measures of microvascular function, indicated by BF and VC responses both before and during 60 seconds of cPLM, which may further corroborate the notion of time and regional differences in vascular recovery throughout the vascular tree.

Inflammation that develops in return of an active immune response may have sweeping implications through systemic circulation (Guo et al., 2020). The pro‐inflammatory mediators released in the cytokine storm have the ability to cross the blood–brain barrier and increase the activation of the sympathetic nervous system (Pongratz & Straub, 2014), as evidenced in the acute phase of SARS‐CoV‐2 infection (Stute, Stickford, Province, et al., 2021). This increase in sympathetic activity can have detrimental effects on several physiologic systems, including alterations in cardiac contraction (Moreira et al., 2017), and impairments in vascular function (Thijssen et al., 2006). Further, autonomic deficits have been described among patients with SARS, 6 months after infection (Lo et al., 2005), providing further evidence for long‐lasting autonomic impairments among those infected with coronaviruses. While peripheral hemodynamic changes were not observed over time, there were significant changes in some central hemodynamic measures. A significant increase in SV AUC_60_ was observed at M4, whereas, ΔpeakCO did not statistically decrease over the 6 months. These results may indicate the central hemodynamics were overcompensating for the decreased vascular function to produce a similar peripheral hyperemic response to cPLM. Following months of recovery, this central hemodynamic compensation may not be needed. Likewise, muscle afferents, stimulated during the cPLM, can increase HR, SV, and CO (Venturelli et al., 2014, 2017), as our current results indicate. These factors, in addition to the NO release, can contribute to the increased perfusion of the dilated vascular bed (Gifford & Richardson, 2017; Ives et al., 2016; Trinity et al., 2010). These alterations in autonomic and cardiovascular measures indicate a disruption in neurovascular control and can contribute to the change in central hemodynamic measures, and lack of vascular alterations.

### 4.5 Markers of oxidative stress, inflammation, nitric oxide bioavailability, and antioxidant levels

#### 4.5.1 Oxidative Stress

The chronic effects of oxidative stress have implications for arterial health, NO bioavailability (Moncada et al., 1991), and vascular function (Eskurza et al., 2004). Elevated oxidative stress among individuals acutely infected by coronaviruses has become increasingly apparent and may be a primary contributor to SARS‐CoV‐2 complications and prolonged recovery (Delgado‐Roche & Mesta, 2020; Suhail et al., 2020). Elevated MDA levels have been observed in many disease states including aged adults (Mutlu‐Turkoglu et al., 2003), and have been observed to even be approximately six times higher in COVID patients 4 months post‐infection compared with both hypertensive patients and a control group (Lambadiari et al., 2021), suggesting lipid peroxidation may persist for several months after infection. Interestingly, our currently reported TBARS levels across the first 6 months following SARS‐CoV‐2 infection in young adults appear to be approximately five times higher than our previously reported TBARS levels for young healthy adults (Augenreich et al., 2020), suggesting lipid peroxidation may persist for several months even among young adults with mild symptoms. Likewise, elevated protein carbonyl levels may be associated with numerous disease states (Dalle‐Donne et al., 2003; Huang et al., 2013; Johnson & Travis, 1979; Totan et al., 2009) and with age (Gil et al., 2006; Mutlu‐Turkoglu et al., 2003). In the current investigation, we did not observe a change to either protein carbonyl despite being ~6 times higher than our previously reported healthy young adults (Augenreich et al., 2020), which may be an indication of a lack of improvement in protein oxidative stress among young adults with mild symptoms of SARS‐CoV‐2 during the first 6 months following infection. While quarantine procedures prevented earlier observations than 3–4 weeks after SARS‐CoV‐2 infection in the current investigation, significant elevations in oxidative stress during the first weeks of infection may be even higher than the levels observed here, which has been theorized to be central to the acute symptoms caused by SARS‐CoV‐2 (Laforge et al., 2020). Regardless, these persistently higher levels of lipid and protein oxidative stress compared to previously reported healthy young adults may be deleterious for the peripheral vasculature and could increase the risk of cardiovascular disease progression (Siti et al., 2015) which should be considered even among those with mild symptoms after SARS‐CoV‐2 infection.

#### 4.5.2 Inflammation

Coronaviruses, such as SARS‐CoV and SARS‐CoV‐2, provoke the production and release of pro‐inflammatory cytokines (Barnes et al., 2020). Elevated IL‐6 levels may be associated with adverse clinical outcomes (Coomes & Haghbayan, 2020; Kermali et al., 2020) including cardiovascular complications (Ridker et al., 2000), and may depend on disease severity (Chen et al., 2020; Gao et al., 2020; Henry et al., 2020; Huang et al., 2020; McGonagle et al., 2020; Ponti et al., 2020; Ruan et al., 2020; Wu et al., 2020; Zhang et al., 2020; Zhou et al., 2020). A meta‐analysis of IL‐6 concentrations in hospital patients with COVID‐19 revealed levels approximately three times higher in patients with more severe COVID‐19 complications, compared with patients suffering from other, “non‐complicated,” diseases (Coomes & Haghbayan, 2020). Furthermore, persistent inflammation, noted by high concentrations of several circulating cytokines, is seen in patients with COVID‐19 through 2 months post‐hospital discharge (Montefusco et al., 2021). However, more work is surely needed to confirm these preliminary observations.

Similar to IL‐6, CRP is a strong indicator of the presence and severity of COVID‐19 infection (Gao et al., 2020; Kermali et al., 2020), as well as a strong predictor for the risk of cardiovascular events (Avan et al., 2018; Ridker et al., 2000) and mortality (Ruan et al., 2020). CRP concentrations have been shown to be significantly elevated in the initial stages of infection in hospitalized COVID‐19 patients (Ali, 2020; Mandal et al., 2021) as well as at hospital discharge, but they have been observed to return to healthy levels 4 to 6 weeks following hospital discharge (Mandal et al., 2021). This current investigation observed consistent, low concentrations of CRP both acutely, and throughout recovery compared to our previously reported in healthy young adults (Augenreich et al., 2020). Given the complexity of the inflammatory cascade, observational timing is critical to observe changes in the multitude of inflammatory mediators. Recently, IL‐10, an anti‐inflammatory mediator which may act in a negative feedback loop within the inflammatory cascade and also correlates with disease severity (Han et al., 2020), was observed to be significantly elevated only days after SARS‐CoV‐2 infection but prior to any patient symptoms (Trinity et al., 2021), suggesting the timing of the inflammatory cascade may vary between subjects and with varying disease severity.

### 4.6 Antioxidants/nitric oxide bioavailability

Reactive oxygen species (ROS) play various roles in regulating vascular function (Wolin, 2009). Excess production of ROS can cause a decrease in NO bioavailability, resulting in endothelial damage and subsequent vascular dysfunction (Suematsu et al., 2002; Wolin, 2009), vasoconstriction, inflammation, and redox imbalance (Beltran‐Garcia et al., 2020). Surprisingly, circulating SOD levels have been observed to be elevated in a disease‐severity manner among those hospitalized with COVID‐19 (Mehri et al., 2021), which may be a remedial mechanism to counteract the effects of high oxidative stress. Interestingly, our results of consistent levels of SOD over time may be intended to defend against consistently high oxidative stress levels. It has been speculated antioxidant capacity may be more impacted during the early phases of infection (Laforge et al., 2020). Therefore, future investigations should focus on both acute as well as longer term impacts on antioxidant capacity. Further, NO has been shown to be negatively impacted by SARS‐CoV‐2 infection, regardless of disease severity, with individuals showing reduced nitrite levels 4 months after hospitalized treatment and discharge (Wang et al., 2021). Indeed, NO has been shown to exert inhibitory effects on SARS‐CoV‐2 (Stefano et al., 2020) and may even be a therapeutic target for those recovering from SARS‐CoV‐2, as clinical trials of inhaled NO underway (Ignarro, 2020). While the current investigation provides evidence of consistent nitrite levels months after SARS‐CoV‐2 infection among young adults with a mild symptom, there will continue to be an urgent need to gauge the long‐term implications of alterations or lack thereof in NO bioavailability on peripheral vascular function as it pertains to lasting health decrements caused by SARS‐CoV‐2 (Marshall, 2020).

### 4.7 Limitations

We recognize several limitations to this real‐world, observational investigations, including the use of oral contraceptives in most of our female participants (n = 6). Although contraceptive use was consistent throughout the testing period, it could impact vascular function (Friedman et al., 2011). Despite the diurnal variation of vascular function remaining contentious (Bau et al., 2008; Jarvisalo et al., 2006; Kim et al., 2015), subjects were encouraged and primarily attended similar testing times across visits, although this was not always possible, and some visit start times may have varied ~4 h throughout the day. Although vascular function alterations within the menstrual cycle may be minimal (Williams et al., 2020), female participants reported to the lab at 1‐month intervals which minimized menstrual cycle fluctuations in vascular function within participants, while the cycle phase between participants was not controlled for. Our participant pool consisted of otherwise healthy, young adults, free of any cardiovascular and pulmonary diseases, and exhibited no‐to‐mild symptoms from the infection. Advancing age and the presence of chronic medical conditions are widely known to be associated with the disease severity of COVID‐19 infection (Jordan et al., 2020; Zhou et al., 2020). Those with preexisting comorbidities have more severe symptoms and higher mortality (Figliozzi et al., 2020). A limitation of this real‐world data is the inability to collect data prior to SARS‐CoV‐2 infection. Further, a time control group was not possible during this early, fluid stage of the COVID‐19 pandemic (Szeghy & Ratchford, 2022). Many SARS‐CoV‐2 cases are undetected, and more than half of patients may have an initial false negative PCR test (Arevalo‐Rodriguez et al., 2020), making it difficult to determine if control participants have been infected with the SARS‐CoV‐2 virus before or especially during longitudinal testing. Vascular alterations may even predate positive SARS‐CoV‐2 testing (Trinity et al., 2021), making longitudinal testing of a control group inappropriate during an active pandemic. While this investigation was the first to longitudinally investigate the impact of SARS‐CoV‐2 in the proceeding months after infection, more work is needed across a broad range of populations and among those with more severe illnesses. Certainly, future studies can learn from these initial findings when choosing time points, patient populations, and sample sizes.

## 5 CONCLUSION

This study was the first to longitudinally track peripheral vascular function in the arms and legs as well as the circulating biomarkers are known to regulate vascular function in young adults recovering from SARS‐CoV‐2 for 6 months after initial infection. Among mild cases of SARS‐CoV‐2 in young adults, the vascular function required several months to recover, which was unrelated to circulating markers of inflammation, oxidative stress, antioxidant capacity, and NO bioavailability. Together, these results suggest persistent ramifications for cardiovascular health, even among those recovering from mild illness and among otherwise healthy, young adults with SARS‐CoV‐2 which should be critically considered as the impact of SARS‐CoV‐2 in the general population continues.

## AUTHOR CONTRIBUTIONS

J.L.S., A.S.L.S., and S.M.R. conceived and designed the research; V.M.P., R.E.S, N.L.S, M.A.A., C.E.B., J.L.S., A.S.L.S, and S.M.R. performed the experiments; V.M.P., R.E.S., N.L.S, M.A.A., C.E.B., J.L.S., A.S.L.S., and S.M.R. analyzed the data; V.M.P., R.E.S., N.L.S., M.A.A., C.E.B., J.L.S., A.S.L.S., and S.M.R. interpreted the results of experiments; V.M.P. and R.E.S. prepared the figures; V.M.P., R.E.S., and S.M.R., drafted the manuscript; V.M.P., R.E.S. N.L.S., M.A.A., C.E.B., J.L.S., A.S.L.S., and S.M.R. edited and revised the manuscript; V.M.P., R.E.S., N.L.S., M.A.A., C.E.B., J.L.S., A.S.L.S., and S.M.R. approved final version of manuscript. All authors have read and approved the final version of this manuscript and agree to be accountable for all aspects of the work in ensuring that questions related to the accuracy or integrity of any part of the work are appropriately investigated and resolved. All persons designated as authors qualify for authorship, and all those who qualify for authorship are listed.

## FUNDING INFORMATION

This study was partially supported by an internal COVID‐19 Research Cluster Award at Appalachian State University.

## CONFLICT OF INTEREST

The authors have no competing interests to declare.

## ETHICS STATEMENT

All procedures were approved by the Appalachian State University Institutional Review Board (IRB_20–0304, IRB_20–0151) in accordance with the ethical standards described by the Declaration of Helsinki. Prior to testing, experimental procedures were explained, both in writing and verbally, and participants provided written informed consent.
